# Comparative radiological features of disseminated disease due to *Mycobacterium tuberculosis *vs non-*tuberculosis *mycobacteria among AIDS patients in Brazil

**DOI:** 10.1186/1471-2334-8-24

**Published:** 2008-02-29

**Authors:** Rodrigo P dos Santos, Karin L Scheid, Denise MC Willers, Luciano Z Goldani

**Affiliations:** 1 Section of Infectious Diseases, Hospital de Clínicas de Porto Alegre, Universidade Federal do Rio Grande do Sul, Brazil

## Abstract

**Background:**

Disseminated mycobacterial disease is an important cause of morbidity and mortality in patients with HIV-infection. Nonspecific clinical presentation makes the diagnosis difficult and sometimes neglected.

**Methods:**

We conducted a retrospective cohort study to compare the presentation of disseminated Mycobacterial tuberculosis (MTB) and *non-tuberculous Mycobacterial *(NTM) disease in HIV-positive patients from 1996 to 2006 in Brazil.

**Results:**

Tuberculosis (TB) was diagnosed in 65 patients (67.7%) and NTM in 31 (32.3%) patients. Patients with NTM had lower CD4 T cells counts (median 13.0 cells/mm^3 ^*versus *42.0 cells/mm^3^, P = 0.002). Patients with tuberculosis had significantly more positive acid-fast smears (48.0% *vs *13.6%, P = 0.01). On chest X-ray, miliary infiltrate was only seen in patients with MTB (28.1% *vs*. 0.0%, P = 0.01). Pleural effusion was more common in patients with MTB (45.6% *vs*. 13.0%, P = 0.01). Abdominal adenopathy (73.1% *vs*. 33.3%, P = 0.003) and splenic hypoechoic nodules (38.5% *vs*. 0.0%, P = 0.002) were more common in patients with TB.

**Conclusion:**

Miliary pulmonary pattern on X-ray, pleural effusion, abdominal adenopathy, and splenic hypoechoic nodules were imaging findings associated with the diagnosis of tuberculosis in HIV-infected patients. Recognition of these imaging features will help to distinguish TB from NTM in AIDS patients with fever of unknown origin due to disseminated mycobacterial disease.

## Background

The use of highly active antiretroviral (HAART) therapy has been associated with a marked decrease in the incidence of opportunistic disease in patients with AIDS [1,2]. The incidence of non-tuberculous mycobacteria especially *Mycobacterium avium complex *(MAC) infections, the most common mycobacterial disease among immune compromised patients in developed countries [3,4], has dramatically declined since 1995 because of HAART [1,5]. For *Mycobacterial tuberculosis *(MTB) infections, this reduction was not so dramatic [6], but observational studies have demonstrated a protective effect of HAART in patients infected with MTB [7,8].

Universal access to antiretroviral medications has reduced the risk of tuberculosis in patients with AIDS in developing countries such as Brazil [9,10]. However, infections with MTB have reached endemic levels and continue to be a major public health problem in these countries [11,12]. Although previous mycobacterial infection has been associated with protection against subsequent mycobacterial infection [13], this is not always the rule [14]. On the other hand, the diagnosis of *non-tuberculous mycobacterial *(NTM) disease is still neglected in these settings considering its variable prevalence [3,4,13,15]. In Brazil, the rate of NTM disease can vary from 0% to 45% [11,16-18]. *Mycobacterium avium *complex is the major NTM specie identified in Brazil [19].

Rapid laboratorial detection of *Mycobacterial *species is difficult due to its dependence on the growth of usually slow growing mycobacteria. Speciation of a *Mycobacterium *isolate using these standard methods is a lengthy process based on subjective data interpretation. Commercially available molecular methods for speciation represent a useful and promising tool but are expensive and do not cover the number of mycobacteria that can be reliably identified in clinical laboratories. In addition, diagnosis based solely on the clinical presentation is unreliable considering that most HIV-infected patients with disseminated disease present systemic symptoms, such as fever, weight loss, and anorexia [20,21].

Abdominal and chest imaging are the initial steps in the investigation of fever of unknown origin (FUO) [22]. Considering that mycobacterial infections are the most common cause of FUO in patients with AIDS [23,24], radiological manifestations might be an important clue for the early diagnosis of tuberculosis (TB) or NTM disease in HIV-infected patients.

The goal of our study was to determine the most common radiological findings in patients with AIDS and disseminated mycobacterial disease caused by NTM and MTB in a tertiary care-hospital in Brazil.

## Methods

We performed a retrospective cohort study at Hospital de Clínicas de Porto Alegre, a 735-bed accommodation tertiary-care hospital located in southern Brazil. From 1996 to 2006 all patients with the diagnosis of HIV infection and disseminated mycobacterial disease were included in the study. Patients were selected by the review of data from the records of microbiology laboratory.

Disseminated disease was characterized when we had a positive identification of MTB or NTM in either one of the following cultures: blood, bone marrow, or liver biopsies. For isolation of *Mycobacterium *sp. a radiometric system (BACTEC 460 TB – Becton & Dickinson Diagnostic Instrument Systems, Sparks, MD, USA), and non-radiometric systems (BACTEC 9240 – Becton & Dickinson Diagnostic Instrument Systems, Sparks, MD, USA; and, BacT/Alert 240 – bioMérieux, Marcy-lEtoile, France) were used. For identification of MTB or NTM, *NAP *(*p-nitro-alfa-acetylamino-beta-hydroxypropiophenone*) test was performed [25,26]. This test is highly specific for the identification of Mycobacterium tuberculosis complex (99%) [27]. In cases which *NAP *test could not be done, the identification of species were made by the visualization of the aspect of the colony – presence or absence of cord formation in liquid media – due to the high sensitivity and specificity of this method [28,29]. We did not use DNA probes for speciation of mycobacteria.

We included for analysis, the most recent CD4 lymphocyte count and HIV viral load test in a six-month period before the hospital arrival.

The imaging studies included – chest X-ray, abdominal ultrasound or computerized tomography (CT) – referred only to exams performed during the first 21 days of the patient's hospital stay.

A descriptive analysis of all the variables collected from each patient was performed. The chi-squared test or Ficher's exact test was used for univariate analysis of selected variables. Associations were considered statistically significant when P value was < 0.05. Data were compiled using Epi-Info 6.04 version and analyzed in SPSS 11.5 version program.

The ethic committee of Hospital de Clínicas de Porto Alegre approved the study. The authors signed a written commitment to guarantee the confidentiality of the data retrospectively collected.

## Results

One-hundred and seven patients with disseminated mycobacterial disease were identified from 1996 to 2006. Eleven patients were excluded from the comparative analysis: six (5.6%) for incomplete data registry, three (2.8%) for simultaneous diagnosis of TB and NTM disease, and two (1.9%) because of lack of species identification. From the 96 remaining patients, disseminated TB was diagnosed in sixty-five patients (67.7%), and thirty-one (32.3%) had NTM disease.

The characteristics of patients with TB and NTM are shown in Table 1. A lower CD4 lymphocyte count was associated with NTM infection. Black race was associated with the diagnosis of TB. Patients with NTM were more exposed or were on use of HAART than patients with TB (57.6% versus 25.8%; P = 0.004). There was no significant difference between TB group and NTM group with respect to any other parameter. None of the patients were on MAC chemoprophylaxis. And none of the patients had the diagnosis of IRIS (immune reconstitution inflammatory syndrome).

**Table 1:** Demographic characteristics of the HIV-infected patients with disseminated Mycobacterial disease

	All Patients^1 ^(n = 96)	MTB (n = 65)	NTM (n = 31)	*P*
Age	32.0	32.0	34.0	.43
Men	71 (74.0)	49 (75.4)	22 (71.0)	.83
Race^2^				.05
White	63 (66.3)	39 (60.9)	24 (77.4)	
Black	23 (24.2)	20 (31.3)	3 (9.7)	
Other	9 (9.5)	5 (7.8)	4 (12.9)	
HIV exposure				.34
Sexual	46 (57.5)	28 (52.8)	18 (56.7)	
IV drug use	34 (42.5)	25 (47.2)	9 (33.3)	
Previous ART	35 (36.4)	17 (25.8)	18 (57.6)	.004
Median CD4 (cels/mm^3^) (No. of patients)	(53)	42.0 (34)	13.0 (19)	.002
Median viral load (log/mL) (No. of patients)	5.2 (11)	4.7 (5)	5.4 (6)	.83
Anti-HCV positive	26 (35.6)	18 (37.5)	8 (32.0)	.83
HBsAg positive	6 (8.8)	4 (8.5)	2 (9.5)	.74

Note. Data are no. (%) of patients, unless otherwise indicated. MTB, *Mycobacterium tuberculosis; *NTM, *non-tuberculous mycobacteria; *IV, intravenous; ART, antiretroviral therapy.

^1 ^Patients diagnosed with tuberculosis and *non-tuberculous mycobacteria *infection.

^2 ^P value related to black and white race groups comparison.

Our study confirms the high sensitivity and specificity of the presence of cord formation [28,29]. Considering the culture as the gold standard, sixty-four of 65 patients with TB had the presence of cord formation (sensitivity of 98%) and 30 of 31 patients with NTM disease had non-cord formation (specificity of 96%) by the visualization of the aspect of the colony.

There was no statistical significant difference comparing the clinical presentation in both groups of patients. The most common clinical symptoms were as follows: weight loss (92.7%), fever (89.6%), respiratory symptoms (75.0%), such as cough, hemoptysis, shortness of breath, and chest pain, and gastrointestinal manifestations (68.8%).

Only 27 patients had positive results on acid-fast smear on sputum or bronchoalveolar lavage, of these, twenty-four (48.0%) in the TB group and three (13.6%) in NTM infection group (P = 0.01).

The chest films and abdominal ultrasound or CT findings are shown in Table 2 and 3, respectively. The chest X-ray was normal in ten (13.2%) patients, while in abdominal image the result was normal in five (7.7%). There was no difference in terms of presence or absence of positive findings on chest X-ray or abdominal imaging comparing the patients with MTB or NTM disease. Seven patients (24.0%) with NTM infection and five (8.0%) patients with tuberculosis had normal chest X-ray findings (P = 0.07). One patient (4.5%) with NTM and three patients (5.5%) with MTB infection had normal abdominal findings on ultrasound or CT (P = 0.68).

**Table 2:** Chest X-ray abnormalities in HIV-infected patients with disseminated mycobacterial disease

	All Patients^1 ^(n = 80)	MTB (n = 57)	NTM (n = 23)	*P*
Diffuse infiltrate	41 (51.3)	28 (49.1)	13 (56.5)	.72
Consolidation	30 (37.5)	21 (36.8)	9 (39.1)	.92
Pleural effusion	29 (36.3)	26 (45.6)	3 (13.0)	.001
Adenopathy	25 (31.3)	21 (36.8)	4 (17.4)	.15
Miliary infiltrate	16 (20.0)	16 (28.1)	0 (0.0)	.001
Atelectasis	3 (3.8)	2 (3.5)	1 (4.3)	.63
Cardiomegaly	3 (3.8)	2 (3.5)	1 (4.3)	.63
Cavitation	3 (3.8)	3 (5.3)	0 (0.0)	.63
Nodules	3 (3.8)	3 (5.3)	0 (0.0)	.63

Note. Data are no. (%) of patients, unless otherwise indicated. MTB, *Mycobacterium tuberculosis; *NTM, *non-tuberculous mycobacteria*.

^1 ^Patients diagnosed with tuberculosis, and *non-tuberculous mycobacteria *infection.

**Table 3:** Abdominal imaging by ultrasound or abdominal CT in HIV-infected patients with disseminated mycobacterial disease

	All patients^1 ^(n = 73)	MTB (n = 52)	NTM (n = 21)	*P*
Adenopathy	45 (61.6)	38 (73.1)	7 (33.3)	.003
Hepatomegaly	30 (41.1)	19 (36.5)	11 (52.4)	.32
Ascities	25 (34.2)	18 (34.6)	7 (33.3)	.86
Splenic hypodensities	20 (27.4)	20 (38.5)	0 (0.0)	.002
Hepatosplenomegaly	20 (27.4)	15 (28.8)	5 (23.8)	.88
Biliary tract alterations	19 (26.0)	12 (23.1)	7 (33.3)	.54
Splenomegaly	18 (24.7)	13 (25.0)	5 (23.8)	.84
Liver echotexture alteration	13 (17.8)	7 (13.5)	6 (28.6)	.86
Liver hypodensities	8 (11.0)	5 (9.6)	3 (14.3)	.74
Pancreatic lesions	3 (4.1)	3 (5.8)	0 (0.0)	.63
Intestinal abnormalities	2 (2.7)	2 (3.8)	0 (0.0)	.90
Abdominal Abscess	1 (1.4)	1 (1.9)	0 (0.0)	.63

Note. Data are no. (%) of patients, unless otherwise indicated. MTB, *Mycobacterium tuberculosis; *NTM, *non-tuberculous mycobacteria.*

^1 ^Patients diagnosed with tuberculosis, and *non-tuberculous mycobacteria *infection.

The most common findings on chest X-ray of HIV-infected patients with disseminated mycobacterial disease were pulmonary interstitial infiltrate (51.3%), consolidation (37.5%), pleural effusion (36.3%), and adenopathy (31.3%). On abdominal image, adenopathy (61.6%), hepatomegaly (41.1%), ascites (34.2%), and splenic hypodense lesions (27.4%) were the most common abnormalities described. Micronodular infiltrate was identified in sixteen patients (28.1%) with MTB infection and in none with NTM disease (P = 0.01). Pleural effusion was more common in patients with TB (45.6% *vs *13.0%. P = 0.01). Other chest X-ray abnormalities were similar in both groups. Abdominal adenopathy was more frequent in patients with disseminated TB (73.1%, n = 38) than patients with NTM disease (33.3%, n = 7; P = 0.003). Only patients with MTB infection presented splenic hypodense lesions (38.5%; n = 20; P = 0.002). Other abdominal findings were associated with neither TB nor NTM infection.

When patients were stratified for the lowest CD4 lymphocyte count strata (CD4 ≤ 50 cels/mm^3^), all the imaging abnormalities previously associated to the diagnosis of disseminated TB (miliary infiltrate, pleural effusion, abdominal adenopathy, and splenic hypodense nodules) remained statistically significant for this diagnosis.

## Discussion

We identified relevant imaging features that could help to distinguish TB from NTM in patients with AIDS and disseminated mycobacterial disease. The presence splenic hypoechoic nodules or micronodules suggestive of splenic microabscesses on abdominal ultrasound and CT scan were highly specific for TB in HIV-patients with disseminated mycobacterial disease, considering it was not seen in patients with NTM infection. Studies have demonstrated a higher prevalence of splenic lesions in HIV-infected patients with disseminated TB compared with those with disseminated NTM [30,31]. Bernabeu-Wittel et al have shown, in 36 patients with HIV infection and splenic microabscesses, that TB and NTM were the aetiology of 43% and 15% of patients respectively [30]. Radin found that 30% of patients with tuberculosis and 7% of patients with MAC had focal lesions in the spleen [31].

Previous non-comparative studies [32,33] have shown the association between TB and abdominal adenopathy. Monill-Serra, for instance, found that out of 76 patients with TB, twenty-seven had the presence of retroperitoneal and mesenteric adenopathy on sonographic imaging. Although some authors have advocated that virtually 100% of HIV-infected patients with MAC presented abdominal adenopathy [31,34,35], in our population the presence of adenopathy were predominantly seen in HIV-infected patients with TB.

Considering intrathoracic adenopathy, Jasmer et al [36] evaluated the imaging features of 318 HIV-infected patients, mostly with disseminated mycobacterial disease. The majority of HIV-infected patients (74%) diagnosed with TB had intrathoracic adenopathy on chest CT scan compared with 50% of the patients with NTM infection. The data did not demonstrate statistically significant difference in our patients, probably because we evaluated only the chest X-rays in contrast to CT scan, which has a higher sensitivity for the detection of intrathoracic adenopathy.

Miliary infiltrate was highly associated with diagnosis of TB. Considering that our HIV-infected patients with NTM did not present miliary pattern on chest X-ray. Hsieh et al [37] have found that none of their HIV-infected patients with MAC presented miliary pattern on chest X-ray. However, three HIV-infected patients with TB presented with miliary infiltrate on chest x-ray.

In patients with AIDS, atypical radiographic presentation of TB in severe immunossuppressed patients seems to occur frequently, and an interstitial diffuse infiltrate is one of the most common radiographic abnormality [37], as described in our patients. Significant pulmonary involvement has not been seen commonly in disseminated MAC infection [38], and a miliary pattern on X-ray is rarely seen in these patients [39-42]. It has been mostly reported in patients with infection by non-tuberculous mycobacteria other than MAC such as patients with *Mycobacterium xenopi *infection [43]. However, consolidations or nodular infiltrates, interstitial infiltrate, pulmonary mass and cavitation have been occasionally reported in HIV-infected patients with MAC infection [42]. In patients with AIDS and disseminated mycobacterial infection, the presence of pulmonary disease and miliary changes on X-ray strongly suggests the diagnosis of TB.

We have found that pleural effusion was associated with the diagnosis of TB. In contrast to disseminated MAC disease, pleural effusions have been frequently described in patients with disseminated TB. In fact, Hsieh et al, found no patient with MAC and pleural effusion in their study [37].

Infections due to MAC are related to more immunossupressed states [44,45]. Whereas MTB infections can affect patients with higher levels of CD4 counts, and is associated with a greater inflammatory response and granuloma formation. In our study, HIV-infected patients with disseminated TB had a higher median CD4 lymphocyte count compared with patients with disseminate NTM. However when we controlled our results for the lowest CD4 count strata (CD4 < 50 cels/mm^3^), all the imaging differences associated with the diagnosis of TB remained statistically significant. Some have advocated that the manifestations of TB in abdomen are related to the immunologic state of the patient [34,35], our data suggest that even in the lowest strata of CD4 counts, patients with TB still seem to have more exuberant disease presentation with abdominal adenopathy and granuloma formation than NTM disease.

Of interest, the sensitivity of acid-fast smears for the diagnosing disseminated disease was low in our patients with disseminated NTM and tuberculosis mycobacteria. Smith et al. have shown that positive acid-fast smears were frequently found in patients with disseminated tuberculosis disease and HIV (96%), and there was no difference related to HIV-status or level of immunossupression to the yield of acid-fast sputum smears [46]. Samb et al. evaluated factors associated with negative sputum smears in patients with disseminated TB and pulmonary involvement: absence of cavitation, lack of cough, HIV seropositivity, CD4 count above 200 cels/mm^3^, and age over 40 years was associated with smear negativity [47]. In disseminated MAC infection, the sensitivity of acid-fast smears and cultures from the respiratory tract is characteristically low [48]. Besides the low sensitivity for diagnosing TB and MAC in HIV-infected patients, the specificity of the acid-fast smear for the diagnosis of TB in our study was 83%, compared to patients with NTM disease.

## Conclusion

In summary, miliary pulmonary pattern, pleural effusion, abdominal adenopathy, and splenic hypoechoic nodules were imaging findings associated with the diagnosis of disseminated *tuberculosis *in HIV-infected patients. Although the retrospective source of data, our results emphasize the importance of imaging studies as valuable tool to help differentiate MTB and NTM disseminated infections in HIV-infected patients, considering the similar clinical presentation of both diseases. Recognition of these imaging features can lead to a tentative diagnosis so that appropriate therapy can be instituted before the results of mycobacterial cultures become available.

## Competing interests

The author(s) declare that they have no competing interests.

## Authors' contributions

RPS: conceived of the study, participated in the design of the study, participated in data collection and wrote the manuscript.

KLS: participated in data collection and helped to draft the manuscript.

DMCW: helped to draft the manuscript.

LZG: conceived of the study, participated in the design and coordination of the study, helped to draft the manuscript.

All authors read and approved the final manuscript.

## Pre-publication history

The pre-publication history for this paper can be accessed here:


